# Development of a prediction nomogram for 1-month mortality in neonates with congenital diaphragmatic hernia

**DOI:** 10.1186/s12893-024-02479-z

**Published:** 2024-06-27

**Authors:** Zhong Feng, Yandong Wei, Ying Wang, Chao Liu, Dong Qu, Jingna Li, Lishuang Ma, Wenquan Niu

**Affiliations:** 1 Department of Neonatal Surgery, Children’s Hospital Capital Institute of Pediatrics, No.2 Yabao Rd., Chaoyang District, Beijing, 100020 China; 2 Graduate School of Chinese Academy of Medical Sciences & Peking Union Medical College, Beijing, China; 3 Department of Critical Care Medicine, Children’s Hospital Capital Institute of Pediatrics, No.2 Yabao Rd., Chaoyang District, Beijing, 100020 China; 4 Center for Evidence-Based Medicine, Capital Institute of Pediatrics, No.2 Yabao Rd., Chaoyang District, Beijing, 100020 China

**Keywords:** Congenital diaphragmatic hernia, Neonate, 1-month mortality, Prediction, Nomogram

## Abstract

**Objectives:**

Although many prognostic factors in neonates with congenital diaphragmatic hernia (CDH) have been described, no consensus thus far has been reached on which and how many factors are involved. The aim of this study is to analyze the association of multiple prenatal and postnatal factors with 1-month mortality of neonates with CDH and to construct a nomogram prediction model based on significant factors.

**Methods:**

A retrospective analysis of neonates with CDH at our center from 2013 to 2022 was conducted. The primary outcome was 1-month mortality. All study variables were obtained either prenatally or on the first day of life. Risk for 1-month mortality of CDH was quantified by odds ratio (OR) with 95% confidence interval (CI) in multivariable logistic regression models.

**Results:**

After graded multivariable adjustment, six factors were found to be independently and consistently associated with the significant risk of 1-month mortality in neonates with CDH, including gestational age of prenatal diagnosis (OR, 95% CI, *P* value: 0.845, 0.772 to 0.925, < 0.001), observed-to-expected lung-to-head ratio (0.907, 0.873 to 0.943, < 0.001), liver herniation (3.226, 1.361 to 7.648, 0.008), severity of pulmonary hypertension (6.170, 2.678 to 14.217, < 0.001), diameter of defect (1.560, 1.084 to 2.245, 0.017), and oxygen index (6.298, 3.383 to 11.724, < 0.001). Based on six significant factors identified, a nomogram model was constructed to predict the risk for 1-month mortality in neonates with CDH, and this model had decent prediction accuracy as reflected by the C-index of 94.42%.

**Conclusions:**

Our findings provide evidence for the association of six preoperational and intraoperative factors with the risk of 1-month mortality in neonates with CDH, and this association was reinforced in a nomogram model.

**Supplementary Information:**

The online version contains supplementary material available at 10.1186/s12893-024-02479-z.

## Introduction

Congenital diaphragmatic hernia (CDH) is a severe and rare birth defect, with an incidence rate of 2.3:10 000 [1]. CDH is mainly caused by the incomplete development of fetal diaphragm during pregnancy and herniation of abdominal organs into the thoracic cavity, resulting in abnormal lung development and persistent pulmonary hypertension (PH). Currently, surgical repair of diaphragm is the fundamental treatment of choice for CDH. Although many patients meet operation conditions and undergo surgical repair, the mortality rate of neonatal severe CDH remains as high as 70% [2, 3], which makes the therapeutic effect and long-term prognosis of CDH a challengeable task. Therefore, early identification of high-risk pediatric patients with CDH by providing active and effective management strategies will be of great significance to improve its prognosis.

Prior studies have identified some factors responsible for CDH survival, such as prenatal ultrasound markers, gestational age at diagnosis, birth weight, concomitant deformity, and postpartum blood gas index [4–6]. However, given the highly heterogeneous nature of CDH, the relative risk attributable to a single factor may be small. To improve prediction accuracy, a variety of evaluation models have been developed by incorporating multiple postpartum factors, including score of Neonatal Acute Physiology-version II (SNAP-II), the CDH study group (CDHSG) probability of survival equation, the Wilford Hall/Santa Rosa clinical prediction formula (WHSRPF), and the Brindle score [7–10]. However, although the diagnosis and treatment of CDH is closely linked between obstetrics and pediatrics, few studies have assessed its clinical course by simultaneously analyzing the prenatal and postnatal factors [11]. It is hence necessitated to establish a more comprehensive clinical prediction model to enhance assessment accuracy and risk stratification of neonates with CDH.

To fill this gap in knowledge and yield more information, we aimed to analyze demographic features, clinical characteristics, outcomes of CDH liveborn infants from a tertiary perinatal center in Beijing to identify potential factors that were associated with 1-month mortality and create a nomogram model to enhance prediction performance.

## Methods

### Study design

The study was retrospective in design, and it was conducted in the Department of Neonatal Surgery, Children’s Hospital of Capital Institute of Pediatrics in Beijing, China. Approval was obtained from the ethics committees of this hospital. Study procedures followed approved research protocols.

### Eligible criteria

The medical records of neonates with CDH who were treated in our department between September 2013 and December 2022 were reviewed. Inclusion criteria were as follows: (1) definite imaging diagnosis of CDH by prenatal diagnostic centers (nationally certified prenatal diagnosis institutions) in Beijing; (2) transferred to our hospital immediately after diagnosis through green channel; (3) neonates with respiratory distress who required invasive ventilation support after birth; Exclusion criteria were as follows: (1) incomplete medical records or follow- up data; (2) not being treated for the first time in our hospital after birth; (3) postnatal diagnosis of CDH.

### Study children

In China, pregnant women routinely undergo their first ultrasound examination at 6–8 weeks to confirm that the fetus is in the uterine cavity and alive, and meanwhile to observe the fetal heartbeat and embryonic sprout. Afterwards, ultrasound examinations are typically performed every 4–6 weeks, and the standard number of ultrasound examinations throughout pregnancy is usually 6–8 times. In addition, the National Health Commission of China has stipulated that fetal ultrasound examinations must include ultrasound examination in early pregnancy (11–13 weeks) and screen for major structural malformations in the second trimester (20–24 weeks). If a suspicious congenital malformation is detected during prenatal screening, the pregnant woman will be referred to a nationally certified prenatal diagnosis center for confirmation and regular follow-ups.

Total 142 neonates with prenatal diagnosis were included in this study. All neonates were born in obstetrics department of the tertiary general hospital or specific obstetrics and gynecology hospital in Beijing, and were transported to the Neonatal Intensive Care Unit (NICU) in our hospital under mechanical ventilation. Because most maternity hospitals are separated from pediatric hospitals in China, our department, formed by a mature multidisciplinary team, has established a green channel, which can facilitate fast and efficient fetal consultation and neonatal transportation from obstetric departments. The whole process of diagnosis and treatment was carried out together with neonatal surgery, obstetrics, pediatric cardiac surgery, intensive care medicine, imaging, anesthesiology, and neonatology departments to provide optimal and individualized therapeutic regimens for neonates with CDH.

The entire treatment process mainly followed the EURO Consortium guidelines (2015) and Chinese Guidelines for Clinical Management of Fetal CDH (2022). Gentle ventilation with permissive hypercapnia before surgery and strict fluid management were adopted. Vasoactive drugs including dopamine, dobutamine, and epinephrine were administered in neonates with circulatory dysfunction. PH of CDH was minimized through standard use of iNO, Sildenafil, and Treprostinil [12]. The preoperative conditions of neonates with CDH were evaluated comprehensively together with blood gas, ventilator parameters, and echocardiography. Surgical repair was performed after achieving physiological stability, and thoracoscopic surgery was preferred. In our department, due to qualification limitations, neonates with CDH had no access to extracorporeal membrane oxygenation (ECMO) and fetoscopic endoluminal tracheal occlusion (FETO).

### Data collection

Primary outcome was appraised within 30 days after birth. Data from neonates on possible risk of 1-month mortality were collected via medical record system. Demographic and clinical data analyzed in this study included: gender, birth weight, delivery mode, gestational age (GA) at birth, GA of prenatal diagnosis (CDH found on the first ultrasound screening), observed-to-expected lung-to-head ratio (o/e LHR), heart structural abnormalities, affected side, liver position, diameter of diaphragmatic defect measured by postnatal ultrasound, and optimal oxygen index (OI) in the first postnatal day. The OI was calculated using the optimal blood gas results within the first postnatal day as inhaled oxygen concentration (%) × mean airway pressure (cmH_2_O, 1cmH_2_O = 0.098 kPa) / oxygen partial pressure (mmHg), as previously reported [13]. Severity of PH was determined through tricuspid regurgitation (TR) velocity and patent ductus arteriosus (PDA) flow patterns on echocardiography. According to the Bernoulli equation, right ventricular systolic pressure (RVSP) calculated from TR velocity can be approximately equal to pulmonary artery systolic pressure (PASP). A moderate and severe PH was defined as estimated PASP ≥ 45 or 2/3-fold systemic systolic pressure [14, 15]. All postnatal variables were measured within 24 h after birth.

### Statistical analysis

Data processing and statistical analyses were completed using the STATA software (version 14.0, Stata Corp, TX) unless otherwise indicated. Two-sided *P* value < 0.05 was considered statistically significant. Based on 1-month survival outcome, all study neonates were categorized into two groups, viz. survival group and non-survival group.

Continuous data are expressed as mean (standard deviation) or median [interquartile range], and categorical data as number (percentage). Between-group comparisons were implemented by t-test, rank-sum or χ^2^ tests, where appropriate.

To identify factors in significant association with CDH, univariate Logistic regression analyses were first done without considering any confounders and then adjusting for sex, GA at birth, and delivery mode, and additionally for birth weight, affected CDH side, and cardiac anomalies. Effect-size estimates are expressed as odds ratio (OR) and 95% confidence interval (95% CI).

Predictive accuracy obtained by adding significant factors into the basic model was appraised from both calibration and discrimination aspects. Calibration statistics included Akaike information criterion (AIC), Bayesian information criterion (BIC), and the Hosmer-Lemeshow test. From the discrimination aspect, net reclassification index (NRI), integrated discriminant improvement (IDI), and area under receiver operating characteristics (AUROC) to were used to see whether prediction performance was improved. In addition, net benefits for adding significant factors were inspected by decision curve analysis.

A risk prediction nomogram model was established by the R programming environment (version 4.3.0) “rms” package, and prediction accuracy was reflected by concordance index (C-index). Finally, the model was internally validated using 1000 bootstrapping resamples.

## Results

### Baseline characteristics

From September 2013 to December 2022, total 154 neonates with CDH were treated for the first time in our hospital. Nine cases of postpartum diagnosis, 1 case with chromosomal abnormalities, 1 case with no ventilation, and 1 case with incomplete data were excluded. Finally, 142 neonates with prenatal diagnosis of CDH were included in our study. The baseline characteristics of study neonates are shown in Table 1. Total 16 children had heart anomalies, including 7 cases of atrial septal defect (ASD), 2 cases of ventricular septal defect (VSD), 1 case of ASD combined with VSD, 1 case of pulmonary artery stenosis, and 5 cases of surgically treated PDA with large diameter. There were 97 neonates who had survived within 30 days after birth, accounting for 68.3% (97/142).

**Table 1:** Demographic characteristics of neonates with CDH by 1-month mortality status

Characteristics		Overall	Survived	Dead	*p*
Sample size		142	97	45	
Sex (%)	Female	64 (45.1)	44 (45.4)	20 (44.4)	1.000
	Male	78 (54.9)	53 (54.6)	25 (55.6)	
Delivery (%)	Caesarean birth	115 (81.0)	76 (78.4)	39 (86.7)	0.345
	Eutocia	27 (19.0)	21 (21.6)	6 (13.3)	
GA of prenatal diagnosis(weeks)		25.00	27.00	23.00	< 0.001
		[23.00, 31.00]	[24.00, 32.00]	[22.00, 25.00]	
GA at birth (weeks)		37.50	37.50	37.25	0.103
		[37.00, 38.10]	[37.10, 38.20]	[36.00, 38.00]	
Birth weight (kg)		3.00	3.02	2.80	0.071
		[2.60, 3.31]	[2.76, 3.32]	[2.32, 3.20]	
o/e LHR		50.83 (15.27)	55.71 (14.23)	40.32 (11.81)	< 0.001
Liver herniation (%)	False	93 (65.5)	72 (74.2)	21 (46.7)	0.002
	True	49 (34.5)	25 (25.8)	24 (53.3)	
Cardiac anomalies (%)	False	126 (88.7)	85 (87.6)	41 (91.1)	0.776
	True	16 (11.3)	12 (12.4)	4 (8.9)	
CDH affected side (%)	Left	110 (77.5)	79 (81.4)	31 (68.9)	0.147
	Right	32 (22.5)	18 (18.6)	14 (31.1)	
Severity of PH	Trivial and mild	83 (58.5)	70 (72.2)	13 (28.9)	< 0.001
	Moderate and severe	59 (41.5)	27 (27.8)	32 (71.1)	
Diameter of defect (cm)		3.75	3.50	4.00	0.002
		[3.00, 4.00]	[3.00, 4.00]	[3.50, 5.00]	
OI		4.02	2.96	17.62	< 0.001
		[2.51, 10.42]	[2.31, 5.03]	[8.53, 28.10]	

Abbreviations: CDH, congenital diaphragmatic hernia; GA, gestational age; o/e LHR, observed-to-expected lung-to-head ratio; PH, pulmonary hypertension; OI, oxygen index. Data are expressed as mean (standard deviation), count (percentage) or median [interquartile range] where appropriate

### Identification of significant factors

Correlation plot was made to visualize pairwise relations of continuous variables (Fig. S1). The correlation between GA at birth and birth weight was relatively high, with a correlation coefficient of 0.67. The variance inflation factor (VIF) of all continuous variables was less than 5.

Both forward and backward logistic regression analyses were used to identify potential factors in significant association with the risk for 1-month mortality in neonates with CDH. As shown in Table 2, six factors, including GA of prenatal diagnosis (fully adjusted OR, 95% CI: 0.845, 0.772–0.925), o/e LHR (0.907, 0.873–0.943), liver herniation (3.226, 1.361–7.648), OI (6.298, 3.383–11.724), Severity of PH (6.170, 2.678–14.217), and diameter of defect (1.560, 1.084–2.245) were found to be associated with the significant risk of 1-month mortality in neonates with CDH before and after adjusting for gender, gestational weeks at birth, and birth weight, delivery mode, affected side, and cardiac anomalies (all *P* < 0.05).

**Table 2:** Identification of potential factors in significant association with 1-month mortality of CDH neonates

Variables	OR	95% CI	*P*
Before adjustment			
GA of prenatal diagnosis	0.840	0.769–0.917	< 0.001
o/e LHR	0.913	0.881–0.946	< 0.001
Liver herniation	3.291	1.568–6.910	0.002
Severity of PH	6.382	2.917–13.96	< 0.001
Diameter of defect	1.528	1.088–2.147	0.015
OI	6.341	3.474–11.574	< 0.001
**Partial adjustment**			
GA of prenatal diagnosis	0.844	0.772–0.923	< 0.001
o/e LHR	0.912	0.879–0.946	< 0.001
Liver herniation	3.342	1.561–7.153	0.002
Severity of PH	6.912	3.072–15.553	< 0.001
Diameter of defect	1.546	1.088–2.197	0.015
OI	6.156	3.385–11.197	< 0.001
**Multivariable adjustment**			
GA of prenatal diagnosis	0.845	0.772–0.925	< 0.001
o/e LHR	0.907	0.873–0.943	< 0.001
Liver herniation	3.226	1.361–7.648	0.008
Severity of PH	6.170	2.678–14.217	< 0.001
Diameter of defect	1.560	1.084–2.245	0.017
OI	6.298	3.383–11.724	< 0.001

Abbreviations: OR, odds ratio; 95% CI, 95% confidence interval; CDH, congenital diaphragmatic hernia; GA, gestational age; o/e LHR, observed-to-expected lung-to-head ratio; PH, pulmonary hypertension; OI, oxygen index

### Prediction accuracy assessment

**Fig. 1:**
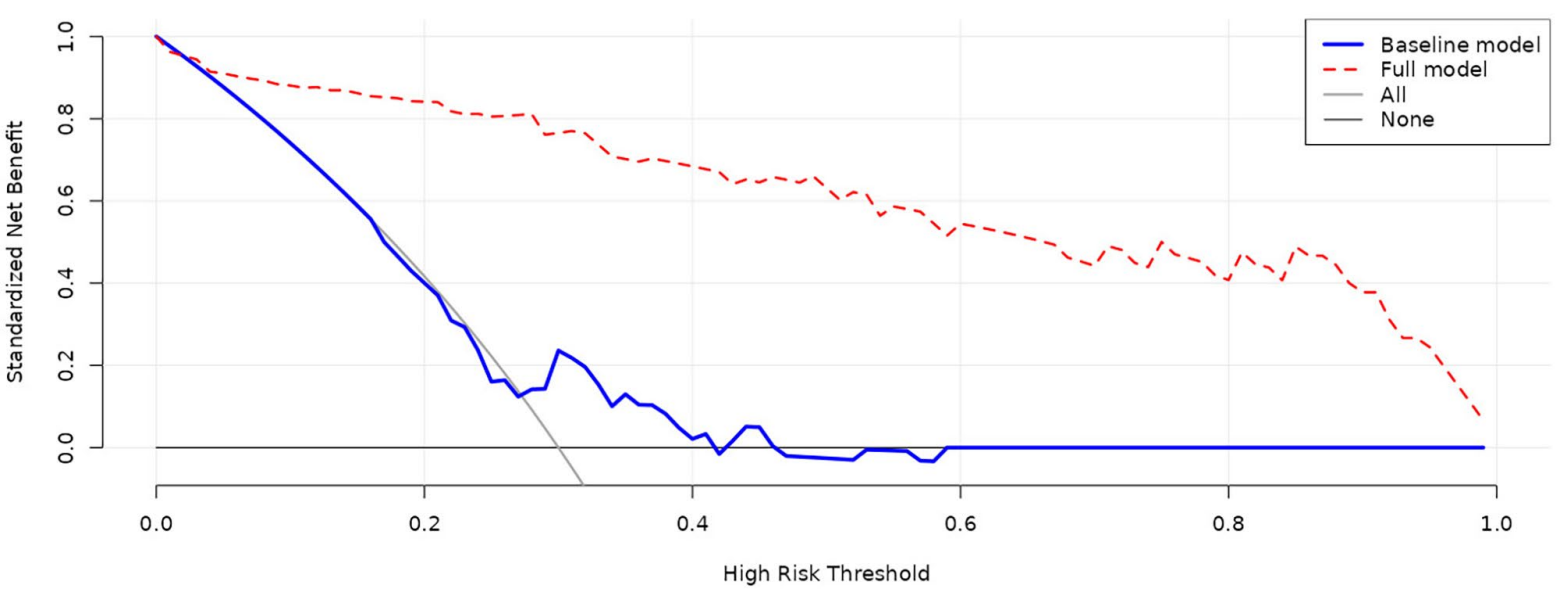
Decision curve analysis on net benefits gained by adding the six significant factors for 1-month mortality in neonates with CDH. The solid grey line represents the net benefit for all patients. The blue curve represented net benefit in the baseline model, and the red curve represented the net benefit in full model by adding six significant factors. The net benefit rate of the full model is higher than the baseline model

To assess the prediction accuracy of the six significant factors identified, two models were constructed, viz. the basic model and the full model. The full model included all variables in this study, and the basic model included all variables except the six significant factors. Both calibration and discrimination statistics were used to assess the prediction accuracy gained by adding the six significant factors to the basic model (Table 3). The Hosmer-Lemeshow test demonstrated that both models were well fitted (*P* > 0.1). Prediction accuracy was significantly improved in the full model relative to the basic model. As revealed by the NRI, both models differed significantly in prediction performance (*P* < 0.0001). ROC curves of both models were shown in Fig. S2. Decision curve analysis indicated that the net benefits gained by adding the six significant factors to the basic model were obvious (Fig. 1).

**Table 3:** Prediction accuracy gained by adding the six significant factors identified for 1-moth mortality of CDH

Statistics	Basic model	Full model
Calibration		
AIC	182.12	95.66
BIC	193.95	140.0
HL test (P)	0.515	0.923
**Discrimination**		
NRI (P)	< 0.0001	
IDI (P)	< 0.0001	
AUROC	0.594	0.960
AUROC (P)	< 0.0001	

Abbreviations: CDH, congenital diaphragmatic hernia; AIC, Akaike information criterion; BIC, Bayesian information criterion; HL test, Hosmer-Lemeshow test; NRI, net reclassification index; IDI, integrated discriminant improvement; AUROC, area under receiver operating characteristics

### Nomogram model

**Fig. 2:**
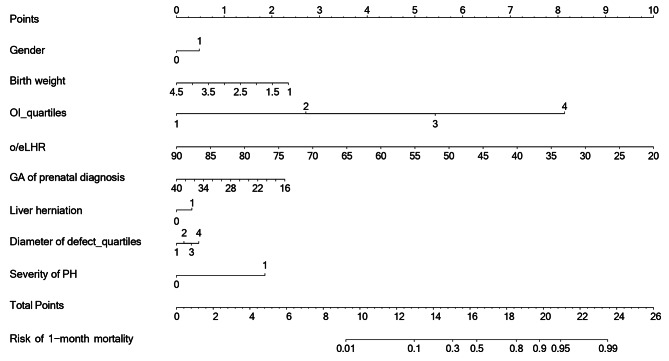
Prediction nomogram for the 1-month mortality risk in neonates with CDH. Abbreviations: CDH, congenital diaphragmatic hernia; GA, gestational age; o/e LHR, observed-to-expected lung-to-head ratio; PH, pulmonary hypertension; OI, oxygen index. According to the clinical characteristics of a CDH neonate, the scores for each independent predictor can be obtained and summed. The total score obtained is projected downward to obtain the probability of 1-month mortality for this neonate. Each factor is assigned a weighted total score, which indicates the probability of 1-month mortality of patients with CDH. Definitions of factors in this nomogram model: for gender, 0 represents female and 1 represents male; for GA of prenatal diagnosis, it ranges within 16–40 weeks; for birth weight, it ranges within 1–4.5 kg; for liver herniation, 0 represents without liver herniation and 1 represents with liver herniation; for o/e LHR, it ranges within 20–90%; for severity of PH, 0 represents trivial or mild PH and 1 represents moderate to severe PH; for diameter of defect (classification by quartiles), 1 represents diameter of defect less than 3 cm, 2 represents diameter of defect equal or greater than 3 cm and less than 3.75 cm, 3 represents diameter of defect equal or greater than 3.75 cm and less than 4 cm, and 4 represents diameter of defect equal or greater than 4 cm; for OI (classification by quartiles), 1 represents OI less than 2.5, 2 represents OI equal or greater than 2.5 and less than 4, 3 represents OI equal or greater than 4 and less than 10, and 4 represents OI equal or greater than 10

To enhance practical application, six significant factors affecting the survival of neonates with CDH were integrated to a nomogram model to help visually predict the risk of 1-month mortality (Fig. 2). In this model, each factor was assigned a weighted score, which indicated the probability of 1-month mortality of neonates with CDH. Overall accuracy of nomogram model was good, as reflected by the C-index (0.9442) and calibration curves (Fig. S3).

For example, assuming a newborn girl (0 point) with moderate to severe PH (2 points), diagnosed at 22 weeks gestation (1.75 points), birth weight of 3.0 kg (1 point), o/e LHR of 55% (5 points), OI of 7.5 (5.5 points), confirmed liver herniation (0.5 points), and 3 cm diaphragmatic defect (0.25 points), the total point of 16 indicates that the probability of 1-month mortality was estimated to be about 40%.

### Internal validation

Because all neonates were enrolled from a single center, internal validation was assessed by the Bootstrap method with 1000 resamples. This model had an accuracy of 85.43% and good discrimination as reflected by AUROC value of 0.91 to predict 1-month mortality.

## Discussion

Despite significant improvement in neonatal surgical techniques and intensive care, the mortality rate of neonates diagnosed with CDH still remains very high. To improve survival outcomes, prognostic factors responsible for neonatal CDH have received wide attention from multidisciplinary medical teams and patient families [16]. The key findings of this study are the identification of six independent factors, including GA of prenatal diagnosis, o/e LHR, liver herniation, severity of PH, diameter of defect, and OI in predicting the significant risk of 1-month mortality in neonates with CDH on the first day of life. Clinical predictive model constructed by combining these multiple predictors can stratify high and low-risk populations and provide the ability to tailor management strategies based on severity.

To enhance the power of prediction models, combining prenatal and postnatal variables is generally preferred. However, due to the low incidence and high heterogeneity of this disease, no consensus has been reached on which variable and how many variables are actually involved in the progression of CDH in neonates [11, 17, 18]. The most widely used variable to stratify lung hypoplasia is prenatal measurement o/eLHR combined with liver position [19]. As one of the independent predictors of CDH death, more recent studies from large CDH treatment centers have confirmed that o/e LHR had good predictive efficacy and stable expression during pregnancy [19–22]. Specifically, the mean AUC of o/eLHR < 25% for predicting mortality in neonates with left-sided CDH was 0.73, with a specificity of 0.95 and a relatively low sensitivity of 0.45 [4]. Above lines of evidence are generally consistent with the results of this study. Other studies have shown that the measurement accuracy of LHR was affected by long learning curve and variability among operators [23, 24]. In this study, neonates with CDH were enrolled from obstetrics departments of tertiary hospitals, which have already established mature multidisciplinary cooperation with our center, guaranteeing standardization of measurement. Moreover, our findings indicated that another prenatal factor, GA of prenatal diagnosis, was significantly associated with 1-month mortality in neonates with CDH. This association has thus far remained a matter of debate. Some researchers have proposed that patients diagnosed before 25 weeks of pregnancy had a significantly higher mortality rate than patients diagnosed after 25 weeks [5, 25], while others argued that GA of prenatal diagnosis had no significant effect on the prognosis of neonates with CDH [26]. This inconsistency could be interpreted by the dual-hit hypothesis. The first hit occurs before diaphragmatic defect, which is the primary lung development abnormality and affects both lungs. The second hit only affects the ipsilateral lung by the abdominal organs herniated into the chest though defective diaphragm [6]. Therefore, earlier GA of prenatal diagnosis can indicate a larger diaphragmatic defect and more contents herniated into the chest cavity, causing worse lung development eventually.

Some researchers believe that actual lung function can only be reliably assessed after birth, that is, when the newborn starts breathing, complete pulmonary circulation is established [27]. As mentioned above, the majority of existing prognostic models are targeted at postnatal stages. For instance, a probability of survival equation for CDH was developed by the CDHSG using the 5-min Apgar score and birth weight [10]. Based on the experience of CDHSG, Brindle et al. [9] developed a clinical prediction model by adding PH, presence of major cardiac anomaly, and chromosomal anomaly as new indicators, and this model has been widely adopted in the literature. A recent study recorded that Brindle or optimized Brindle score had fair predictive power in Chinese patients [28]. However, several studies have pointed out that the measurement of PH and 5-min Apgar score after birth may have poor accuracy due to errors from different measuring personnel [28–30]. In addition, many prenatally diagnosed neonates with CDH were intubated directly after birth, which made the Apgar score missing or inaccurate [28]. Obviously, Brindle score was not suitable to our cohort. We noticed the influence of preoperative physiological indicators on prognosis described in previous observations, and took OI as the main indicator to evaluate preoperative physiological stabilization [13]. Our findings indicated that OI had the best predictive performance across six significance factors identified, consistent with the results of recent studies [29].

Our study also provided insights into postpartum image factors that may affect the survival of neonates with CDH. From clinical aspects, diaphragmatic defect is usually the most intuitive indicator for assessing diaphragmatic development in neonates with CDH, and it can be classified as A to D based on defect sizes during operation [31]. A recent study has showed a significant higher mortality in pediatric patients with large diaphragmatic defects [32]. Patients with C and D defects may be more susceptible to increased stress, pulmonary edema, PH exacerbation, and ventilatory fluctuation potentially, which were associated with repair surgery [33]. Our study found that defect diameter measured by preoperative ultrasound was valuable for predicting 1-month mortality of CDH. In addition, the use of echocardiographic factors to monitor treatment and predict CDH prognosis has been a research focus. Some studies have confirmed that RVSP > 45.5, preoperative PDA right-to-left shunting, and right-to-left dominant velocity time integral (VTI), as a reflection of PH and cardiac dysfunction, were associated with CDH prognosis. Consistent with the results of previous studies, our study confirmed that ultrasound assessment of PH has a good predictive effect, and adding this parameter made the prediction model more comprehensive.

Besides the clear strengths of this study including the simultaneous consideration of multiple common, easy-to-obtain factors to predict 1-month mortality of neonates with CDH, some limitations should be acknowledged in this study. Firstly, this study was retrospective in design and enrolled patients from a single center. The generalizability in clinical applications needs to be further verified by prospective cohort studies. Secondly, since all neonates with CDH in our center did not receive the Extracorporeal Membrane Oxygenation (ECMO), whether our prediction model is suitable to predict mortality of CDH infants in ECMO institutes remains an open question, as only a few hospitals are certified for neonatal ECMO in China. This study involves neonates with CDH from mainland China, and extrapolation to the other racial groups is restricted. Thirdly, as GA of prenatal diagnosis generally depends on each medical system to screen fetuses, a consensus is needed for the sake of generalizability.

Taken together, our findings provide evidence for the association of six prenatal and early postnatal factors with the significant risk of 1-month mortality in neonates with CDH, and this association was reinforced in a nomogram model. Importantly, these factors were easy to obtain with strong specificity, making the model simple and effective. This study is conducive to fine stratified clinical management, and provides a new idea for improving the prediction of CDH survival. For practical reasons, we expect that large-scale, prospective, multicenter randomized studies are needed to confirm the contributory role of this new prediction model in clinical practice.

### Electronic supplementary material

Below is the link to the electronic supplementary material.

Supplementary Material 1

Supplementary Material 2

Supplementary Material 3

## Data Availability

All data generated or analyzed during this study are included in this article. Further inquiries can be directed to the corresponding authors.
