# Supplementary material for: Development of a prediction nomogram for 1-month mortality in neonates with congenital diaphragmatic hernia
Source: BMC Surg. 2024 Jun 27;24:198. doi: 10.1186/s12893-024-02479-z (PMC11210016; doi:10.1186/s12893-024-02479-z)
Supplement: Supplementary file 1 — Supplementary Material 1 [file 12893_2024_2479_MOESM1_ESM.docx]

**
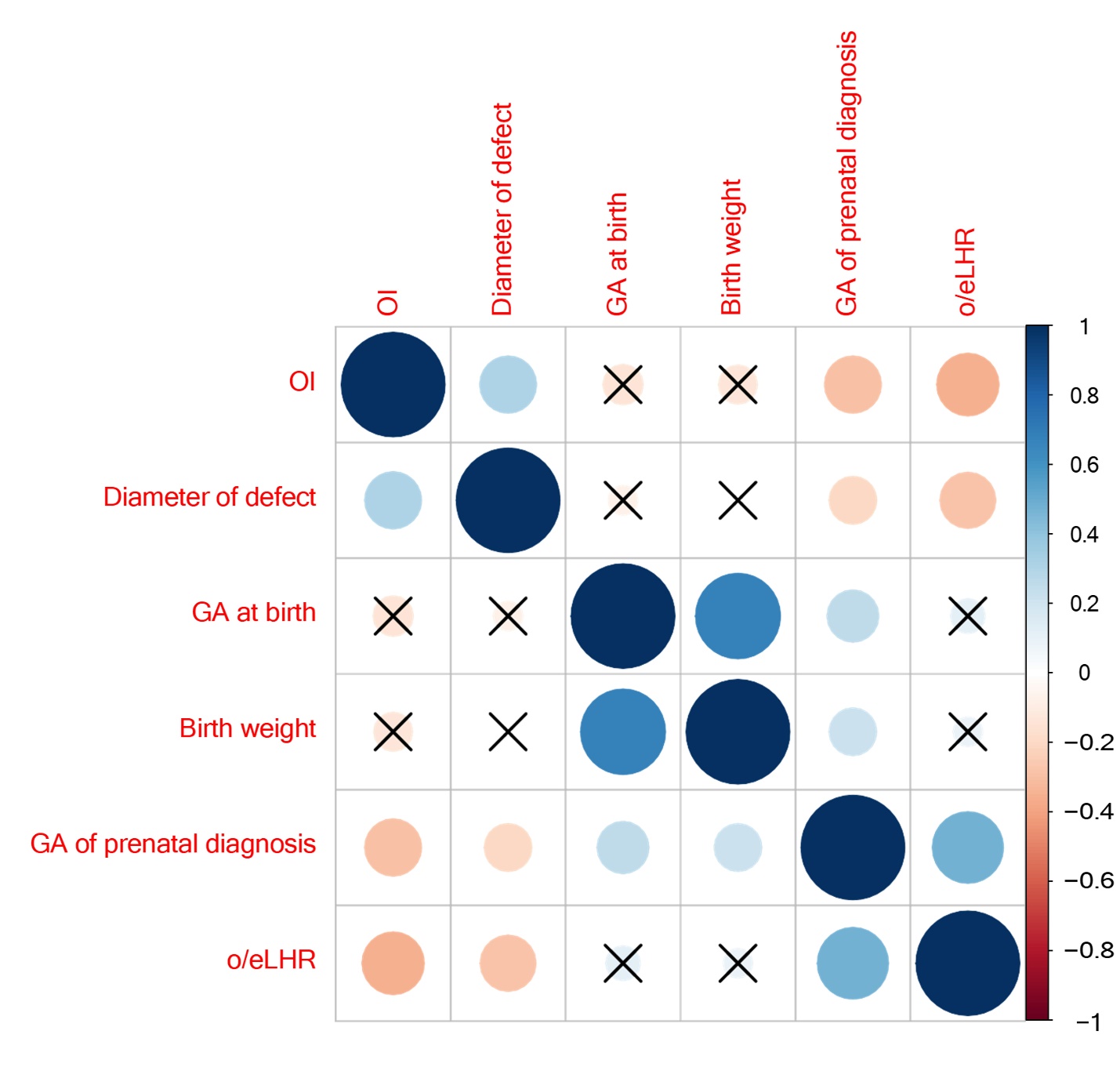
**

**Figure S1.** Correlation plot of continuous variables. The size of the circle represents the correlation coefficient between the two variables. Higher correlation coefficient has larger circle. The color of the circle represents positive or negative correlation. The blue circle represents a positive correlation and the orange circle represents a negative correlation.
