# Supplementary figures and images for: Development of a prediction nomogram for 1-month mortality in neonates with congenital diaphragmatic hernia
Source: BMC Surg. 2024 Jun 27;24:198. doi: 10.1186/s12893-024-02479-z (PMC11210016; doi:10.1186/s12893-024-02479-z)

**
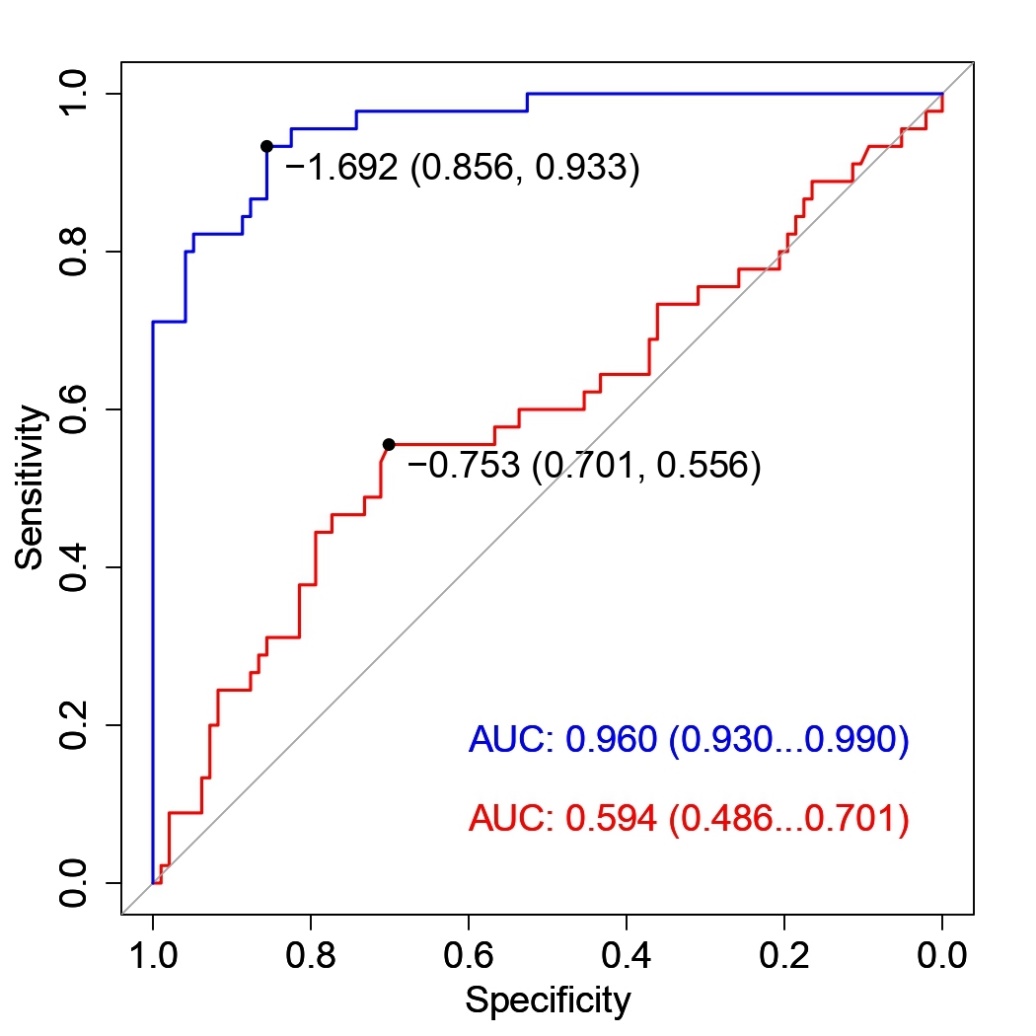
**

**Figure S2.** The area under the curve (AUC) of baseline model (red curve) and full mode (blue curve).

Supplement: Supplementary file 2 — Supplementary Material 2 [file 12893_2024_2479_MOESM2_ESM.docx]
