# Supplementary material for: Development of a prediction nomogram for 1-month mortality in neonates with congenital diaphragmatic hernia
Source: BMC Surg. 2024 Jun 27;24:198. doi: 10.1186/s12893-024-02479-z (PMC11210016; doi:10.1186/s12893-024-02479-z)
Supplement: Supplementary file 3 — Supplementary Material 3 [file 12893_2024_2479_MOESM3_ESM.docx]

**
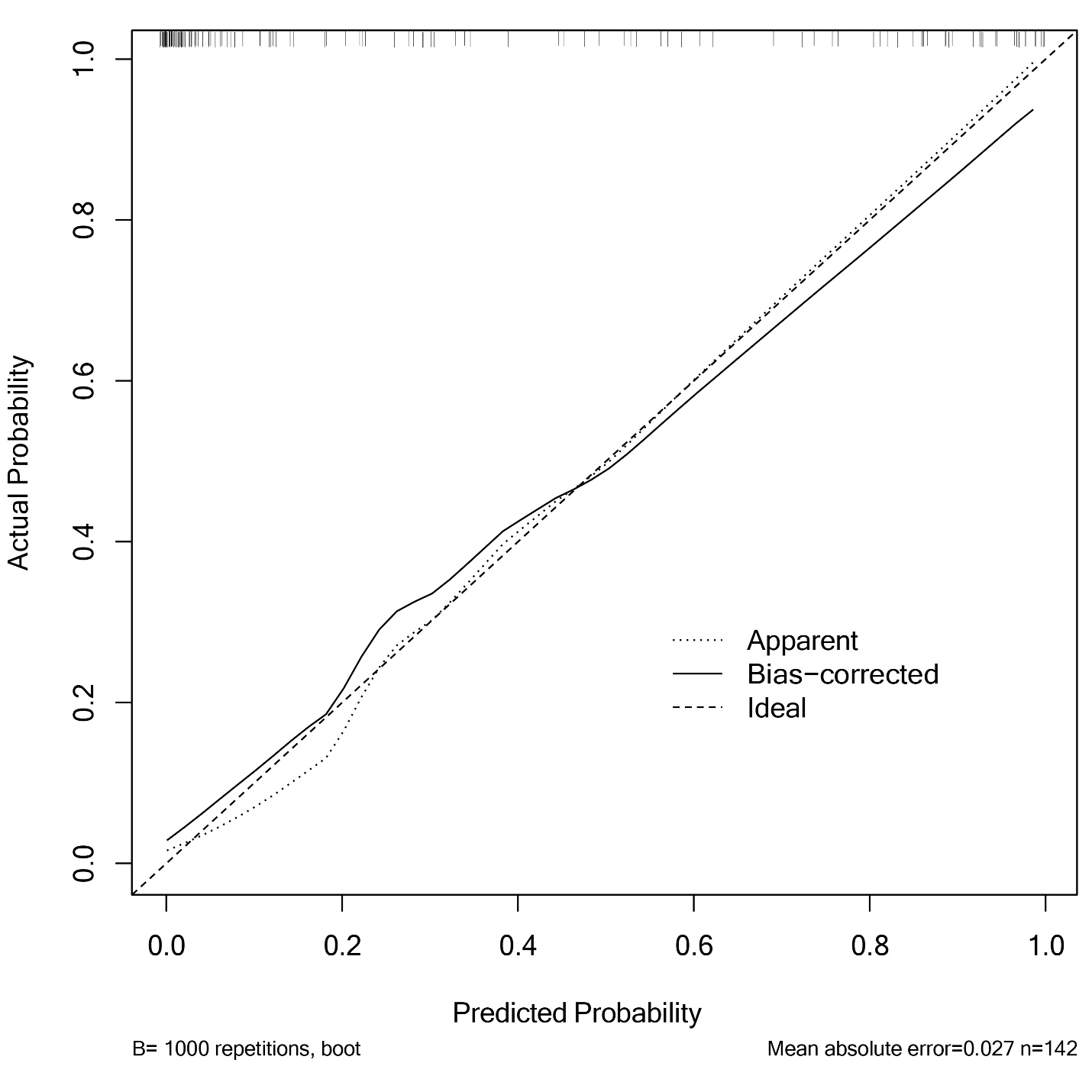
**

**Figure S3.** Calibration curve for the prediction nomogram for 1-month mortality in neonates with congenital diaphragmatic hernia. This curve can evaluate the performance of the nomogram and compare the predicted- and actual-probability of 1-month survival in neonates with congenital diaphragmatic hernia
